# Associations of individual factors and early childhood education and care (ECEC) centres characteristics with preschoolers’ BMI in Germany

**DOI:** 10.1186/s12889-022-13814-5

**Published:** 2022-07-26

**Authors:** Raphael M. Herr, Freia De Bock, Katharina Diehl, Eva Wiedemann, Elena Sterdt, Miriam Blume, Stephanie Hoffmann, Max Herke, Marvin Reuter, Iryna Iashchenko, Sven Schneider

**Affiliations:** 1 Center for Preventive Medicine and Digital Health (CPD), Medical Faculty Mannheim, Heidelberg University, Ludolf-Krehl-Straße 7-11, 68167 Mannheim, Germany; 2 Department of Medical Informatics, Biometry and Epidemiology, Friedrich-Alexander-Universität Erlangen-Nürnberg (FAU), Erlangen, Germany; 3 Child Health Services Unit, Clinic for General Pediatrics, Neonatology and Pediatric Cardiology, Medical Faculty, University of Düsseldorf, Düsseldorf, Germany; 4 Competence Centre for Early Education, Magdeburg-Stendal University of Applied Sciences, Stendal, Germany; 5 Department of Epidemiology and Health Monitoring, Robert Koch-Institute, Berlin, Germany; 6 Department of Public Health, Brandenburg University of Technology Cottbus-Senftenberg, Senftenberg, Germany; 7 Institute of Medical Sociology, Medical Faculty, Martin-Luther-University Halle-Wittenberg, Halle, Germany; 8 Institute of Medical Sociology, Centre for Health and Society, Medical Faculty, University of Düsseldorf, Düsseldorf, Germany; 9 Professorship of Health Economics, Department of Sport and Health Sciences, Technical University of Munich, Munich, Germany

**Keywords:** BMI, Obesity, Preschool children, ECEC centres, Kindergarten, Meso level, Socioeconomic position

## Abstract

**Background:**

The number of obese children is rising worldwide. Many studies have investigated single determinants of children’s body mass index (BMI), yet studies measuring determinants at different potential levels of influence are sparse. The aim of this study is to investigate the independent role of parental socioeconomic position (SEP), additional family factors at the micro level, as well as early childhood education and care (ECEC) centre characteristics at the meso level regarding BMI.

**Methods:**

Analyses used the baseline data of the PReschool INtervention Study (PRINS) including up to 1,151 children from 53 ECEC centres. Multi-level models first estimated the associations of parental SEP indicators (parental school education, vocational training, and household income) with the children’s standard deviation scores for BMI (SDS BMI, standardised for age and gender). Second, structural (number of siblings), psychosocial (strained family relationships), and nutrition behavioural (soft-drink consumption, frequency of fast-food restaurant visits) family factors at the micro level were included. Third, characteristics of the ECEC centre at the meso level in terms of average group size, the ratio of overweight children in the group, ECEC centre type (all-day care), and the location of the ECEC centre (rural vs urban) were included. All analyses were stratified by gender and adjusted for age, migration background, and parental employment status.

**Results:**

Estimates for boys and girls appeared to differ. In the full model, for boys the parental SEP indicators were not related to SDS BMI. Factors related to SDS BMI in boys were: two or more siblings; B = -.55; *p* = 0.045 [ref.: no sibling]), the characteristics of the ECEC centre in terms of average group size (20 – 25 children; B = -.54; *p* = 0.022 [ref.: < 20 children]), and the ratio of overweight children (more overweight children B = -1.39; *p* < 0.001 [ref.: few overweight children]). For girls the number of siblings (two and more siblings; B = .67; *p* = 0.027 [ref.: no sibling]) and average group size (> 25 children; B = -.52; *p* = 0.037 [ref.: < 20 children]) were related to SDS BMI.

**Conclusions:**

The BMI of preschool children appears to be associated with determinants at the micro and meso level, however with some gender differences. The identified factors at the micro and meso level appear largely modifiable and can inform about possible interventions to reduce obesity in preschool children.

## Background

Children’s body mass index (BMI) has been rising over the last decades leading to higher prevalence of weight-related diseases such as overweight and obesity in many high-income countries [1]. Childhood obesity can affect a child’s immediate health, educational attainment and quality of life, and is likely to continue into adulthood, leading to increased risk of negative health outcomes and chronic illness [2]. Therefore, the prevention of paediatric obesity presents a major public health issue and preschool age is considered a "critical window for child development" [3].

Childhood obesity arises from complex interactions among biological, behavioural and socio-environmental factors, including unmodifiable (e.g., genetics, ethnic differences, gestational weight and intrauterine conditions), and modifiable (e.g., socioeconomic position, diet, physical activity, sleep, and parental determinants) factors at different levels [4]. Bronfenbrenner's Ecological Systems Theory [5] offers a comprehensive theoretical framework to identify ecological determinants of health that could be applicable on determinants of obesity among preschool children [6]. It describes a framework through five nested environmental systems in which children interact, spanning from the immediate environment to the interaction of the larger environment. The microsystem reflects the most immediate environment and includes activities and relationships within the family, school, neighbourhood and peers of the children. The mesosystem comprises interconnections between the microsystems, like between the early childhood education and care (ECEC) centre and the parents' home. An exosystem represents a network of relationships to which the child does not belong directly. Examples for an exosystem are mass-media, industry, or local politics. The macrosystem refers to all relationships in a society, including norms, values, conventions and traditions, that influences the development of a child. The chronosystems encompass the temporal dimension of development and transitions over the life course (e.g., going to school). The Ecological Systems Theory thus presents a comprehensive framework for research on determinates of the weight of children at different levels, including the child, family, and childcare setting.

On the micro level, the parental socioeconomic position (SEP) is an established determinant for children’s health status. Previous research has shown that the prevalence of risk factors and diseases, as well as the way in which children cope with illnesses, correlates with SEP. For instance, children with lower-income parents fare worse than those with higher-income parents [7]. Thus, health disparities in physical and mental health such as developmental outcomes and cognitive abilities are evident even among this young age group [8–10]. Social epidemiological studies further indicate that three- to seventeen-year-old children from a lower SEP are more likely to have unhealthy diets, be less physically active and therefore more likely to be obese than their peers from more socially advantaged families [11, 12].

In addition to the SEP, further family factors at the micro level might play a role in child weight, as family is the most proximal environment in preschool children [4, 6]. For example, lifestyle factors associated with obesity like caloric intake and physical inactivity might be learned within the family [4].

Next to these micro level factors such as the parental SEP and individual health behaviour, the importance of institutions at the meso level for health and health inequalities has been increasingly acknowledged in recent years [13]. Besides the family as the primary socialisation institutions, ECEC centres (i.e., kindergartens) are – at least in many high-income countries – the most important socialisation institutions for the group of three- to six-year-old children [14]. In Germany, the attendance rate lies above 92% and every third child under the age of six is cared for full-time [15]. ECEC centres are considered as socialization instances, since – for example – health behaviours such as nutrition and exercise are learned and established in this phase of life and contribute to individual health behaviour in the further course of life [16]. However, existing research on preschool children's health predominantly focuses on the individual level, and a recent review has shown that the number of studies considering meso level characteristics of ECEC centres (i.e., institutional structures) and their association with health, and health behaviours is limited and only very few studies additionally consider the individual SEP [17]. However, there is a reason to assume that the compositional (i.e., aggregated information about the children) and contextual (i.e., structural conditions of an institution) characteristics of ECEC centres at the meso level might add to the explanation of health differences in children [13, 17].

In a recent study, Park et al. [6] identified the socioeconomic background of the child, as well as certain parental perceptions and family factors (pressure to eat, family obesogenic environment, sleep hours, bedtime), and the community factor parents’ perceptions of the family’s physical activity environment independently related to BMI. However, ECEC centre factors were not associated with BMI. One reason might be that associations of individual and meso level factors with BMI might differ for boys and girls. Gender differences were, for example, found in factors related to BMI, like physical activity, watching television, playing video games or participating in sport [18–20]. Furthermore, the association of family circumstance (e.g., siblings, parental education and employment) with television viewing and physical activity also vary by gender, and girls might be more vulnerable to a deprived environment [21, 22].

The aim of this study is to investigate whether and how – in addition to the parental SEP – family factors, as well as meso level factors contribute to explaining BMI levels in children. Therefore, the independent associations of children’s BMI with parental SEP, structural, psychosocial and behavioural family factors, as well as compositional and contextual meso level characteristics of ECECs are estimated in a stepwise approach. To account for the possible differential effects for boys and girls, all analyses were stratified by gender.

## Methods

**Fig. 1:**
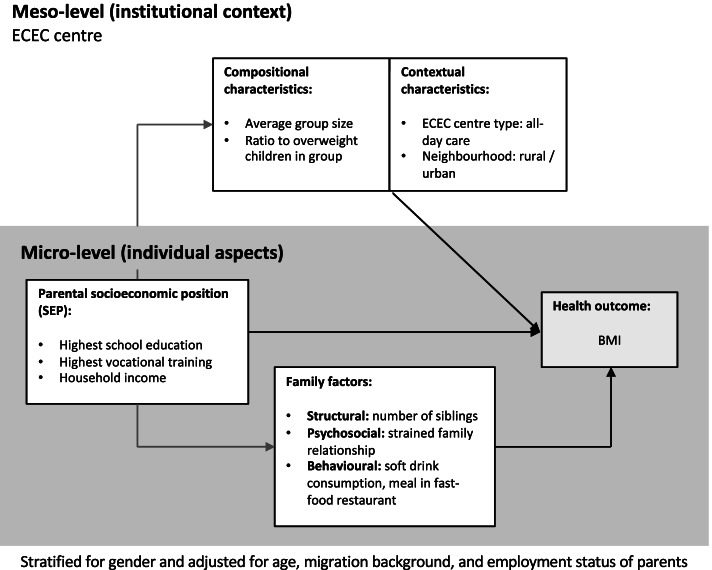
Conceptual model of the study

Figure 1 depicts the conceptual model of our study. Parental SEP is assumed to be related to BMI as a central health indicator. Furthermore, at the individual level, structural, psychosocial and behavioural family factors were considered likely to play a role. Our model also takes into account the meso level in terms of compositional (i.e., the structure of the group) and contextual (i.e., the type and location) characteristics of the ECEC centre.

### Study population

This study used data from the PReschool INtervention Study (PRINS) [23, 24], one of the few studies in Germany that includes information of the ECEC centres visited (meso level) in addition to the individual situation of the children (micro level). PRINS is a cluster-randomised trial on ECEC centre-based interventions into children’s health behaviour. ECEC centres were eligible to participate in the PRINS if they were located in one of three predefined regions in the south of Germany and had applied to participate in the intervention module of a state-funded health promotion programme ‘Komm mit in das gesunde Boot’ (‘Come aboard the health boat’), with at least fifteen children visiting each ECEC centre. The programme was initiated to encourage healthy eating behaviour and physical activity among preschool children with the long-term goal of reducing childhood overweight. Between September 2008 and March 2010, baseline data were assessed in a sample of 1,151 children from 53 ECEC centres. This cross-sectional data prior to the intervention was used for the analyses in this study. Informed written consent was obtained from the parents of the participating children and the Ethics Committee of the Medical Faculty Mannheim at Heidelberg University approved the study (2008-275 N- MA).

### Health outcome

Trained members of the study team visited the 53 ECEC centres to measure – among others – height and weight to assess the BMI of children. Anthropometry measurements followed a standardised protocol [23]. Height was measured to the nearest 0.1 cm (Seca Deutschland, Hamburg, Germany), and weight was measured to the nearest 0.1 kg (Soehnle pharo, Nassau, Germany) in underwear. BMI was calculated by the standard formula (kg/m^2^). Age- and gender-specific BMI z-scores or standard deviation score of the BMI (SDS BMI) were calculated based on the formula presented by Schaffrath Rosario et al. (2010) on representative data for Germany [25]. Compared to the original values, the z-scores are standardized for age and gender and transformed to the value range of a standard normal distribution.

### Micro level: individual and family factors

The parents filled in proxy reports for their children in the form of pre-tested standardised written questionnaires. Parental SEP was used as a proxy for children’s SEP, comprising highest school education, highest vocational training, and household income.

School education was measured by the highest education of the mother or the father, categorized into low (no qualification or secondary school qualification), middle (secondary school qualification to advanced technical college), and high (high school graduation).

Vocational training was assessed by the highest vocational training of the mother or the father and combined in the categories: low (no professional qualification or apprenticeship), middle (vocational, commercial school or technical, master craftsman, technician), and high (technical college degree or university).

Household income was operationalised across the total net household income and was measured in nine categories and summed into low (< 500 Euro to < 2,000 Euro), middle (2,000 to < 3,000 Euro), and high (3,000 to ≥ 4,000 Euro) income.

### Covariates

Age (in months) was indicated by the parents in relation to the question concerning how old the child was at the time of the survey date. Migration background (yes vs no) was assumed if the nationality of the mother or father was not German, the country of birth of the child was not Germany, or the native language of the parents or the language spoken at home was not German. Employment status of the mother and father was measured by the categories full-time, part-time, and not employed (including homemaker, student).

### Structural, psychosocial and behavioural family factors

Information about the number of siblings were categorised into no sibling (only one child in the family), one sibling (two children in the family), or two or more siblings (three or more children in the family)*.* Strained family relationships (yes vs no) were assumed if the parents agreed to one of the following items: major quarrels of the child with the parents, upcoming divorce, divorce, or quarrels with siblings. The self-report from the parent’s questionnaire on the average soft drink consumption of their child was summarised from original categories into no (rarely or not at all) and yes (1–2 glasses / week, 4–6 glasses / week, 1 glass / day, 2–3 glasses / day, 4 or more glasses / day). Visiting fast-food restaurants (yes vs no) was defined when the parents stated that the family has a meal out of home in a fast-food restaurant at least once a week.

### Meso level: Institutional context

For the compositional characteristics, the ECEC centre teachers and institution heads filled in standardised written questionnaires. The average group size was calculated by dividing the number of children in the ECEC centre by the number of groups in the centre, and it was categorised into three groups: < 20 children, 20—25 children and > 25 children. The ratio of overweight children within the ECEC centre was computed by dividing the number of overweight children as indicated by the ECEC centre management by the number of children in the ECEC centre, and it was split into tertiles (few, some, or more overweight children in the group).

In addition to this compositional characteristic, contextual characteristics were also considered at the meso level. The ECEC centre management stated whether the ECEC centre type was all-day care or not. The composition of the ECEC centre also included the surrounding neighbourhood. A structured protocol was applied to categorise ECEC centres’ location as either rural or urban. Satellite views at a predefined altitude were examined independently by two research team members (Google Earth, accessed 6^th^ June 2008). Rural sites were defined as those that had forest, parks and green spaces within the cut-out but no highways or industrial areas. All other preschools were categorised as being located in an urban area. In each case, ratings were compared and differences were discussed until consensus was reached [23].

### Statistical analyses

Descriptive analyses were shown as proportions and number of observations for categorial variables and mean and standard deviation (SD) for continuous variables. Group differences were examined by Chi^2^ tests for categorial variables, and T-tests (two groups) or F-test (more than two groups) for continuous variables, supplemented by Scheffé post-hoc tests.

The associations of the independent variables with SDS BMI as an outcome were estimated by gender-stratified hierarchical random intercept models (multilevel mixed-effects linear regressions) with children at level 1 and ECEC centres at level 2. A stepwise calculation of models based on our conceptual model (cf. Figure 1) was applied. Model 1 comprised the three SEP indicators highest school education, highest vocational training and household income, as well as the control variables age, migration background, and employment status of the mother and father. Model 2 additionally considered the block of the structural, psychosocial, and behavioural family factors. Model 3 further included the meso level in terms of compositional and contextual ECEC centre characteristics. The level of significance was a priori set to *p* < 0.05 and all analyses were performed using StataSE (version 14).

## Results

Of the total 1,151 children from the 53 ECEC centres in Southern Germany, 47.5% were female and the children were on average 57.48 (SD = 9.03) months old (min: 32 months; max: 81 months; Table 1). About one-third of these children had a migration background (36.1%) and four out of ten were a single child at the time of the survey (38.8%). While 7.8% of the mothers were working full-time, 56.4% were employed part-time, and 35.8% were not employed, 94.6% of the fathers were working full-time, 1.9% part-time, and 3.5% were not employed, which is similar to the distribution for West Germany [26]. Regarding the meso level, around one-third of the ECEC centres offered all-day care (35.7%). A group size between 20 and 25 children (56.8%) was most typical. Around one in four ECEC centres was located in an urban location (28.2%). Among the overall cohort, the average BMI measured with standard instruments was 15.32 (SD = 1.57; min: 10.18; max: 27.2), and the SDS BMI was -0.31 (SD = 1.14).

**Table 1:** Study population description

		Total (*n* = 1,151)		Boys (*n* = 604)		Girls (*n* = 546)		Test value	*P*-value
Micro level									
Body-Mass-Index (mean / SD)		15.32	1.57	15.38	1.60	15.25	1.53	1.30	0.194
SDS Body-Mass-Index (mean / SD)		-0.31	1.14	-0.31	1.21	-0.31	1.05	-0.08	0.933
Parental SEP									
School education	Low (% / n)	13.19	127	12.50	63	13.94	64	0.94	0.624
	Middle (% / n)	43.93	423	43.25	218	44.66	205		
	High (% / n)	42.89	413	44.25	223	41.39	190		
Vocational training	Low (% / n)	37.49	346	37.04	180	37.99	166	0.45	0.798
	Middle (% / n)	23.19	214	22.63	110	23.80	104		
	High (% / n)	39.33	363	40.33	196	38.22	167		
Income	Low (% / n)	22.38	184	22.25	93	22.52	91	0.02	0.991
	Middle (% / n)	37.35	307	37.56	157	37.13	150		
	High (% / n)	40.27	331	40.19	168	40.35	163		
Covariates									
Age (months; mean / SD)		57.48	9.03	57.11	9.18	57.88	8.85	-1.46	0.145
Migration background (yes; % / n)		36.07	351	34.97	178	37.15	172	0.50	0.480
Employment status mother	Full time (% / n)	7.79	74	8.45	42	7.08	32	2.29	0.320
	Part-time (% / n)	56.42	536	57.95	288	54.87	248		
	Not employed (% / n)	35.79	340	33.60	167	38.05	172		
Employment status father	Full time (% / n)	94.61	860	93.76	451	95.78	409	1.85	0.397
	Part-time (% / n)	1.87	17	2.08	10	1.41	6		
	Not employed (% / n)	3.52	32	4.16	20	2.81	12		
Intermediate aspects									
Structural: number of siblings	No sibling (% / n)	38.76	274	40.66	148	36.73	126	2.78	0.250
	One sibling (% / n)	39.89	282	40.38	147	39.36	135		
	Two and more siblings (% / n)	21.36	151	18.96	69	23.91	82		
Psychosocial: strained family relationships (yes, % / n)		12.72	124	12.77	65	12.47	58	0.02	0.889
Behavioural (nutrition): soft drink consumption (yes, % / n)		21.90	187	21.04	93	22.87	94	0.42	0.519
Behavioural (nutrition): meal in fastfood restaurant (yes, % / n)		46.49	430	47.35	232	45.39	197	0.35	0.552
Meso level									
Average group size	< 20 children (% / n)	22.84	211	24.23	118	21.33	93	1.95	0.377
	20—25 children (% / n)	56.82	525	56.88	277	56.65	247		
	> 25 children (% / n)	20.35	188	18.89	92	22.02	96		
Ratio to overweight children	Few overweight children (% / n)	43.11	410	43.06	217	43.05	192	0.80	0.671
	Some overweight children (% / n)	32.28	307	33.33	168	31.17	139		
	More overweight children (% / n)	24.61	234	23.61	119	25.78	115		
ECEC centre type (all-day care, % / n)		35.71	411	34.44	208	37.18	203	0.94	0.333
Neighbourhood	Rural (% / n)	71.76	826	72.85	440	70.70	386	0.66	0.418
	Urban (% / n)	28.24	325	27.15	164	29.30	160		

*Test value* T-test for continuous variables and Chi^2^ test for categorial variables, *ECEC* Early childhood education and care, *SDS Body-Mass-Index* Age- and gender-specific standard deviation score of the BMI [25]

The association of SEP indicators with SDS BMI levels could be confirmed in bivariate analysis. The SDS BMI of children decreases with parental school education (mean SDS BMI: low = -0.23, middle = -0.24, high = -0.44, *p* = 0.0303), parental vocational training (mean SDS BMI: low = -0.24, middle = -0.27, high = -0.48, *p* = 0.0172), and household income (mean SDS BMI: low = -0.21, middle = -0.24, high = -0.44, *p* = 0.0348).

Gender-stratified bivariate comparisons revealed that the average BMI of boys (15.38; SD = 1.53; SDS BMI = -0.31; SD = 1.21) was not significantly different from the BMI of girls (15.25, SD = 1.53; SDS BMI = -0.31; SD = 1.05). Regarding the independent variables to be included in the later multivariate multi-level models, there were no further significant differences between boys and girls (Table 1). A migration background was slightly higher among girls (37%) than boys (35%) and the average age was also comparable, with 57 months for the boys and 58 months for the girls. 21% of the boys and 23% of the girls consumed soft drinks and almost the half of the studied population visited fast-food restaurants at least once per week (47% and 45%, respectively).

A comparison of the analytic sample applied in the full adjusted multi-level model (Model 3) with the drop-outs revealed small and, in most cases, not significant differences (Table 2). In the analytic sample, the mean SDS BMI score was slightly higher (-0.17 versus -0.36, *p* = 0.021), more soft drinks were consumed (27.65% versus 19.32%, *p* = 0.007), and more frequently fast-food restaurants were visited (51.89% versus 44.34%, *p* = 0.037). The most pronounced differences were found with regard to the number of siblings and average group size of the ECEC centres. In the analytic sample, most children had one (56.06%) or more (26.14%) siblings, while in the drop-out sample most children had no sibling (51.24%, *p* < 0.001). The average group size in the analytic sample was rather lower than in the drop-out sample (*p* < 0.001).

**Table 2:** Drop out analysis

		Drop-out (*n* = 716)		Analytic sample (*n* = 264)		Test value	*P*-value
Micro level							
Body-Mass-Index (mean / SD)		15.27	1.60	15.46	1.46	-1.70	0.090
SDS Body-Mass-Index (mean / SD)		-0.36	1.17	-0.17	1.04	-2.31	0.021
Parental SEP							
School education	Low (% / n)	13.57	95	12.12	32	1.99	0.370
	Middle (% / n)	44.86	314	41.29	109		
	High (% / n)	41.57	291	46.59	123		
Vocational training	Low (% / n)	38.69	255	34.47	91	5.71	0.058
	Middle (% / n)	21.09	139	28.41	75		
	High (% / n)	40.21	265	37.12	98		
Income	Low (% / n)	24.69	138	17.8	47	5.04	0.080
	Middle (% / n)	36.67	205	38.64	102		
	High (% / n)	38.64	216	43.56	115		
Covariates							
Age (months; mean / SD)		57.24	9.22	58.28	8.30	-1.64	0.101
Migration background (yes; % / n)		36.67	260	34.47	91	0.40	0.525
Employment status mother	Full time (% / n)	7.43	51	8.71	23	1.77	0.413
	Part-time (% / n)	57.73	396	53.03	140		
	Not employed (% / n)	34.84	239	38.26	101		
Employment status father	Full time (% / n)	95.19	614	93.18	246	3.69	0.158
	Part-time (% / n)	2.02	13	1.52	4		
	Not employed (% / n)	2.79	18	5.3	14		
Intermediate aspects							
Structural: number of siblings	No sibling (% / n)	51.24	227	17.8	47	79.86	< 0.001
	One sibling (% / n)	30.25	134	56.06	148		
	Two and more siblings (% / n)	18.51	82	26.14	69		
Psychosocial: strained family relationships (yes, % / n)		11.53	82	15.91	42	3.32	0.068
Behavioural (nutrition): soft drink consumption (yes, % / n)		19.32	114	27.65	73	7.40	0.007
Behavioural (nutrition): meal in fastfood restaurant (yes, % / n)		44.33	293	51.89	137	4.34	0.037
Meso level							
Average group size	< 20 children (% / n)	19.55	129	31.06	82	15.46	< 0.001
	20—25 children (% / n)	58.33	385	53.03	140		
	> 25 children (% / n)	22.12	146	15.91	42		
Ratio to overweight children	Few overweight children (% / n)	42.36	291	45.08	119	4.89	0.087
	Some overweight children (% / n)	31.15	214	35.23	93		
	More overweight children (% / n)	26.49	182	19.7	52		
ECEC centre type (all-day care, % / n)		36.53	324	32.95	87	1.13	0.287
Neighbourhood	Rural (% / n)	70.69	627	75.38	199	2.21	0.137
	Urban (% / n)	29.31	260	24.62	65		

*Test value* T-test for continuous variables and Chi^2^ test for categorial variables. Analytic sample corresponds to Model 3 in multi-level analyses, *ECEC* Early childhood education and care, *SDS Body-Mass-Index* Age- and gender-specific standard deviation score of the BMI [25]

The stepwise models of the associations of parental SEP, covariates, family factors, and ECEC characteristics with SDS BMI are shown in Table 3 for boys and Table 4 for girls. In the first model, considering the parental SEP indicators and the covariates age, migration background, and employment status of the parents, among boys only the covariate regarding the employment status of the father was related to SDS BMI (Model 1: not employed; B = 0.72; *p* = 0.022 [ref.: full time]). For girls, in addition to the covariate of employment status of the father (Model 1: part-time; B = -1.02; *p* = 0.022 [ref.: full time]), the SEP indicator high vocational training was negatively related to SDS BMI (Model 1: B = -0.39; *p* = 0.008 [ref.: low]). The high vocational training became insignificant when the family factors were included in the subsequent model 2 (Model 2: B = -0.33; *p* = 0.094 [ref.: low]). From these family factors, the behavioural factor of having a meal in a fast-food restaurant was also related to the SDS BMI in the subsample of girls, however, the *p*-value was above threshold of significance (Model 2: B = -0.27; *p* = 0.057 [ref.: no meal in a fast-food restaurant]). Including the meso level characteristics average group size, ratio to overweight children, form of the ECEC centres, and neighbourhood (Model 3) reduced the intercept variance and the ICC, indicating that the observations within ECEC centres are not more similar than observations from different ECEC centres and that the included covariates explain the variation between the centres. For boys, after controlling for meso level characteristics of the ECEC centre (Model 3), the covariates for employment status of the mother (Model 3: part-time; B = 0.66; *p* = 0.033 [ref.: full time]) and the migration background (Model 3: B = 0.40; *p* = 0.036 [ref.: no migration background]), and two or more siblings (Model 3: B = 0.55; *p* = 0.045 [ref.: no sibling]) had an independent association with the SDS BMI. In addition, the meso level characteristics of average group size (Model 3: 20 – 25 children; B = -0.54; *p* = 0.022 [ref.: < 20 children]) and ratio of overweight children (Model 3: more overweight children B = 1.39; *p* < 0.001 [ref.: few overweight children]) showed an independent association with SDS BMI. In the full adjusted models (Model 3), for girls also the family factor of number of siblings (Model 3: two and more siblings; B = 0.67; *p* = 0.027 [ref.: no sibling]) and average group size > 25 children (Model 3: 20 – 25 children; B = -0.52; *p* = 0.037 [ref.: < 20 children]) showed an association, while all other variables in the model were not related to SDS BMI.

**Table 3:** Multi-level models for SDS BMI in boys

	Model 1			Model 2			Model 3		
Level and Variable	Coeff	S.E	*P*-value	Coeff	S.E	*P*-value	Coeff	S.E	*P*-value
Micro level									
Intercept	-0.137	0.559	0.806	-0.543	0.677	0.423	-0.640	0.771	0.407
Parental SEP									
School education									
Middle (ref. low)	0.277	0.222	0.212	0.158	0.263	0.550	-0.026	0.302	0.930
High (ref: low)	0.110	0.246	0.655	-0.040	0.290	0.891	-0.324	0.327	0.321
Vocational training									
Middle (ref. low)	-0.119	0.176	0.497	-0.159	0.207	0.444	0.158	0.238	0.507
High (ref: low)	-0.015	0.187	0.936	-0.005	0.236	0.985	0.152	0.266	0.567
Income									
Middle (ref. low)	0.064	0.196	0.745	-0.116	0.281	0.679	0.027	0.306	0.929
High (ref: low)	-0.132	0.215	0.539	-0.143	0.287	0.619	-0.051	0.306	0.867
Covariates									
Age (months)	-0.004	0.008	0.619	0.002	0.010	0.852	-0.007	0.011	0.530
Migration background (yes; ref: no)	0.243	0.145	0.094	0.300	0.180	0.096	0.404	0.193	0.036
Employment status mother									
Part-time (ref: full time)	-0.204	0.235	0.386	0.238	0.304	0.434	0.664	0.311	0.033
Unemployed (ref: full time)	-0.230	0.246	0.350	0.164	0.319	0.608	0.285	0.326	0.382
Employment status father									
Part-time (ref: full time)	-0.802	0.518	0.122	-0.565	0.674	0.402	-0.714	0.790	0.366
Unemployed (ref: full time)	0.724	0.317	0.022	0.088	0.409	0.830	-0.055	0.393	0.888
Intermediate Variables									
Structural / material:									
Number of siblings									
One sibling (ref: no sibling)				0.008	0.209	0.969	0.224	0.228	0.327
Two and more siblings (ref: no sibling)				0.124	0.250	0.619	0.554	0.277	0.045
Psychosocial:									
Strained family relationships (yes; ref: no)				0.107	0.243	0.659	0.039	0.250	0.876
Behavioural (nutrition):									
Soft drink consumption (yes; ref: no)				0.000	0.193	0.999	-0.029	0.214	0.894
Meal in fastfood restaurant (yes. sometimes; ref: no)				0.138	0.165	0.404	0.250	0.177	0.157
Meso level									
Average group size (ref: < 20)									
20—25							-0.542	0.237	0.022
> 25							-0.423	0.303	0.162
Ratio to overweight children (ref: few overweight children)									
Some overweight children							0.398	0.254	0.116
More overweight children							1.392	0.304	< 0.001
ECEC form (all-day care; ref: not all-day care)							0.209	0.279	0.453
Neighbourhood (urban. ref: rural)							-0.337	0.235	0.152
Additional information									
Intercept variance	0.092			0.105			0.000		
Within-group variance	1.399			1.002			0.909		
Log likelihood	-580.7			-262.7			-187.9		
Likelihood (LR) ratio test	3.250		0.036	2.310		0.064	0.000		1.000
ICC	0.062			0.095			0.000		
Number of observations	360			180			137		
Number of groups	53			34			29		

*ECEC* Early childhood education and care

**Table 4:** Multi-level models for SDS BMI in girls

	Model 1			Model 2			Model 3		
Level and Variable	Coeff	S.E	*P*-value	Coeff	S.E	*P*-value	Coeff	S.E	*P*-value
Micro level									
Intercept	0.498	0.476	0.296	0.512	0.709	0.471	-0.095	0.857	0.912
Parental SEP									
School education									
Middle (ref. low)	-0.022	0.191	0.909	0.353	0.247	0.154	0.346	0.286	0.226
High (ref: low)	-0.061	0.212	0.773	0.244	0.277	0.377	0.333	0.327	0.309
Vocational training									
Middle (ref. low)	0.074	0.141	0.601	-0.044	0.182	0.810	0.035	0.222	0.874
High (ref: low)	-0.387	0.146	0.008	-0.333	0.198	0.094	-0.423	0.261	0.105
Income									
Middle (ref. low)	-0.031	0.158	0.843	0.259	0.211	0.220	0.158	0.262	0.547
High (ref: low)	0.029	0.173	0.869	0.217	0.241	0.369	0.122	0.299	0.684
Covariates									
Age (months)	-0.009	0.006	0.132	-0.018	0.009	0.043	-0.012	0.012	0.313
Migration background (yes; ref: no)	0.188	0.118	0.111	0.245	0.163	0.132	0.396	0.210	0.059
Employment status mother									
Part-time (ref: full time)	-0.126	0.219	0.566	0.022	0.317	0.946	0.256	0.390	0.511
Unemployed (ref: full time)	-0.217	0.224	0.333	-0.224	0.333	0.500	0.095	0.405	0.816
Employment status father									
Part-time (ref: full time)	-1.015	0.444	0.022	-0.135	0.550	0.806	-0.487	0.712	0.494
Unemployed (ref: full time)	0.112	0.307	0.716	0.391	0.491	0.426	0.261	0.613	0.671
Intermediate Variables									
Structural / material:									
Number of siblings									
One sibling (ref: no sibling)				0.136	0.223	0.541	0.265	0.272	0.331
Two and more siblings (ref: no sibling)				0.380	0.244	0.120	0.666	0.302	0.027
Psychosocial:									
Strained family relationships (yes; ref: no)				-0.112	0.193	0.562	0.213	0.239	0.373
Behavioural (nutrition):									
Soft drink consumption (yes; ref: no)				-0.188	0.161	0.244	-0.341	0.202	0.092
Meal in fastfood restaurant (yes. sometimes; ref: no)				-0.271	0.142	0.057	-0.232	0.173	0.179
Meso level									
Average group size (ref: < 20)									
20—25							-0.129	0.228	0.573
> 25							-0.524	0.251	0.037
Ratio to overweight children (ref: few overweight children)									
Some overweight children							0.039	0.242	0.873
More overweight children							0.341	0.338	0.312
ECEC form (all-day care; ref: not all-day care)							-0.099	0.227	0.662
Neighbourhood (urban. ref: rural)							-0.118	0.264	0.656
Additional information									
Intercept variance	0.014			0.034			0.000		
Within-group variance	0.907			0.725			0.778		
Log likelihood	-471.1			-217.4			-164.3		
Likelihood (LR) ratio test	0.210		0.324	0.460		0.249	0.000		1.000
ICC	0.015			0.044			0.000		
Number of observations	342			170			127		
Number of groups	52			38			33		

*ECEC* early childhood education and care

## Discussion

Increased body weight in young children represents a significant public health issue, as a representative study has revealed that in Germany more than 15% of the children and adolescents between the age of three and seventeen were overweight and the prevalence of obesity in this age group was about 6%, with a higher prevalence of obesity among boys than girls [27]. Health-related lifestyles, societal ideals regarding body weight, and gender-specific influences such as body composition and hormones are discussed in the literature as potential causes [28].

In our German sample of about 1,000 preschool children, the bivariate comparisons show a social gradient towards a higher BMI for socioeconomically disadvantaged children for the three established SEP indicators. However, if the micro and meso level factors were considered in multivariate multi-level models, the association of SEP with BMI did not remain significant, and we were able to provide initial evidence for the relevance of ECEC centre meso level characteristics for BMI in preschool children. Our study shows that the gender-specific analysis proved to be necessary, as different determinants turned out to be relevant for BMI in boys compared to girls. While for both genders, the ECEC centre characteristics of “average group size” was independently negative related to the BMI, only for boys the characteristics “more overweight children” had a pronounced positive relation to BMI. It can thus be concluded that for the BMI of boys and girls different factors play a particularly relevant role. In literature, gender differences were also reported regarding physical activity and television viewing [19, 21].

According to this study, especially the compositional ECEC centre characteristics seemed relevant for the BMI. Previous studies have mainly examined the age [29, 30] and gender composition [31] of groups. These studies have – for example – shown that the gender composition of the ECEC centre group had a significant impact on the development of boys, but not of girls [32]. Research into the age composition of groups has shown that a wider range of ages might be beneficial regarding children’s learning and development [33]. It is also known that meso level characteristics of facilities are related to children's health; for example, because they might have an influence on children's physical activity behaviour [17]. Research results have shown that children moved more and spend less time sitting if the ECEC centre has created a movement-friendly environment [34]. Especially movable and fixed play equipment, a sedentary environment, the physical activity training, and the physical activity education have been found to influence physical activity behaviour [17]. In our study, larger groups are significantly associated with a lower BMI. One possible reason could be that larger groups are more mobile and are located in larger buildings. In this case, this could encourage individual physical activity (e.g., through group activities, games of catch and games with a ball) and the larger space might encourage more movement.

To the best of our knowledge, the present study is the first to consider the group prevalence of overweight. Several reasons might be assumed why boys in a group with more overweight children had a higher BMI, which appears to be one of the most pronounced effects in this study [35, 36]. Some mechanisms by which social networks influence the development of overweight and obesity in adolescents and adults might also apply to preschool children. One underlying mechanism might be social contagion, whereby the group in which the children are embedded determines their weight or weight influencing behaviours [36]. Children might mimic their peers' behaviour related to both healthy and unhealthy food choices as well as to physical activity and sports participation [37]. Thus, social norms and/or model learning might play a role here, in the sense that higher weight is more likely to be perceived as socially accepted. However, further research on this aspect appears to be necessary.

In addition to compositional characteristics of ECEC centres, contextual characteristics have been considered. Literature suggests a distinct role of exposure duration (duration of care), according to which a more extensive ECEC centre attendance is suggested to reduce health inequalities [38]. On the one hand, a reduction can be expected since shared exposure and social as well as health-promoting measures (such as shared meals, shared exercise opportunities and intervention for developmental deficits) take place. On the other hand, ECEC centres might have indirect health effects; for example, through parental health education by the teachers [38]. In this context, it was discussed whether children from socioeconomically deprived families benefit more or less from resources and support than children from families with a higher SEP [39]. However, interestingly the extent of the daily care time in terms of all-day care did not emerge as a significant factor in our study.

A recent scoping review identified the location of ECEC centres as a relevant factor for health behaviour, health and well-being of young children [17]. A closer look at the health outcomes revealed that location was particularly frequently found to be related to nutrition, physical activity, sedentary behaviour, physical health, development and mental health, but not with further factors like body weight and obesity. Our empirical analysis supports this notion and shows no association between the location of the ECEC centre and BMI.

It can only be speculated why some factors were relevant for boys but not for girls, and vice versa. Thus, we cannot clarify why the association between the SEP indicator high vocational training and BMI was only found in girls in the first multivariate model in this study. This association appeared to be non-significant after controlling for family factors in Model 2 (*p* = 0.094). One explanation might be that a more deprived environment could be especially detrimental for girls. An indication of this idea is provided, for example, by Petterson and Albers who have found that the impact of poverty determined by parental income and parental education on cognitive developmental delays was particularly pronounced for girls [22]. In addition, the role of determinant factors of overweight, energy drink consumption, and physical fitness have been found to depended on children’s gender [40–42]. However, in this study neither soft-drink nor fast food consumption were related to BMI in boys or girls. Furthermore, the ratio to overweight children was only a strong and significant determinant for BMI in boys. It might be speculated that for boys the peer group might be more important. However, another study examining physical activity speculated that for boys intrinsic motivators (e.g., desire to be active), while for girls extrinsic motivators (e.g., parental physical activity) appear more important [19]. Taken together, further research is needed to further understand our findings. Gender-sensitivity seems to be an important approach for further research in this regard.

This study has some limitations to report. The central limitation refers to the operationalisation of the constructs. Because this was a secondary data analysis, the ability to capture the individual and ECEC aspects was limited. Future studies should try to extend the operationalisation of the structural, psychosocial, and behavioural family factors, as well as the contextual and compositional ECEC characteristics and compare different aspects. In addition, the underlying model should be tested and results should be compared regarding different (health) outcomes. Another limitation of this study relates to the sample reduction between the models due to missing values and the risk of being underpowered as a consequence. A larger sample size in future studies can help to confirm the stability of the results and expand the findings to other populations than preschool children in the south of Germany. Further studies should additionally consider the physical activity of the children, as well as parental behaviour, which represents an important determinant for the health behaviour of children, especially in younger age.

## Conclusion

In conclusion, it can be summarized that the BMI of preschool children seems to be related to determinants at the micro and meso level. This study has policy implications, allowing to identify factors related to BMI of children on the individual and meso levels. Some of these factors might be amenable compared to mostly stable factors like the SEP. This knowledge thus can guide potential interventions to reduce overweight in children of preschool age.

## Data Availability

The datasets used and analysed during the current study are not publicly available due to data protection regulations but are available from the corresponding author on reasonable request.

## Abbreviations

BMI: Body mass index
ECEC: Early childhood education and care
PRINS: PReschool INtervention Study
SEP: Socioeconomic position
SDS BMI: Age- and gender-specific standard deviation score of the BMI
